# Editorial: Targeting Human Inflammatory Skin Diseases With Natural Products: Exploring Potential Mechanisms and Regulatory Pathways

**DOI:** 10.3389/fphar.2021.791151

**Published:** 2021-11-15

**Authors:** Bey Hing Goh, Andrei Mocan, Jianbo Xiao, Siau Hui Mah, Wei Hsum Yap

**Affiliations:** ^1^ Biofunctional Molecule Exploratory Research Group (BMEX), School of Pharmacy, Monash University Malaysia, Bandar Sunway, Malaysia; ^2^ College of Pharmaceutical Sciences, Zhejiang University, Hangzhou, China; ^3^ Department of Pharmaceutical Botany, “Iuliu Haţieganu” University of Medicine and Pharmacy, Cluj-Napoca, Romania; ^4^ Laboratory of Chromatography, ICHAT, University of Agricultural Sciences and Veterinary Medicine Cluj-Napoca, Cluj-Napoca, Romania; ^5^ Nutrition and Bromatology Group, Department of Analytical Chemistry and Food Science, Faculty of Food Science and Technology, University of Vigo, Ourense, Spain; ^6^ School of Biosciences, Taylor’s University, Subang Jaya, Malaysia; ^7^ Centre for Drug Discovery and Molecular Pharmacology (CDDMP), Faculty of Health and Medical Sciences (FHMS), Taylor’s University, Subang Jaya, Malaysia

**Keywords:** natural products, skin diseases, inflammation, mechanism of action, signaling pathways

## Introduction

For decades, researchers have explored the potential of natural products with immunomodulatory and antioxidant effects for targeting human skin diseases. Skin diseases can be caused by multiple factors including exposure to allergens, genetic inheritance, as well as autoimmune responses, and they cause major health concerns. The use of natural products is gaining popularity due to their long history of use and as a potential source of biologically active agents with anti-inflammatory, immunomodulatory, antioxidant, and chemo-preventive effects. Thus, natural products are continuously being explored for targeting multiple signaling pathways and acting as potential bioactive agents for targeting skin diseases. In this Research Topic, a total of eleven articles were published, illustrating the immunomodulatory mechanisms and regulatory pathways of diverse natural products and their formulations in targeting human skin diseases.

## Natural Products–Herbal Formulations and Bioactive Agents

There is growing interest in the search for novel, effective and safe herbal formulations derived from combinations of medicinal plants which contain active ingredients with multiple effects. The potential of herbal formulations in dermatological applications for the treatment of psoriasis, atopic dermatitis, contact dermatitis, oral ulcers and many other inflammatory skin diseases were highlighted. Herbal medicines are formulated in various forms, including topical agents such as ointment, creams, and emulsions, as well as in oral forms like capsule, tablet, and soup. Lee et al. reported that Chijabyukpi-tang (CBT), an oriental herbal formula traditionally shown to treat eczema, has shown to improve atopic dermatitis-associated symptoms in 2,4-dinitrochlorobenzene (DNCB) treated mice. Lee et al. demonstrated that Socheongryong-tang (SCRT), a traditional formulation originating from East Asia that has been used for treating respiratory and allergic diseases, reduced dependence on steroidal ointment in patients with mild-moderate atopic dermatitis without any significant toxicity effects. On the other hand, LiangXueJieDu (LXJD), an herbal formula that has been commonly used in China to treat psoriasis vulgaris, was also shown to suppress IMQ-induced psoriasis-like lesions in mice (Zhao et al.). Besides, Jo et al. identified the potential of Derma-H which has been used as traditional herb for treating swelling, dryness, and itchiness, to inhibit skin hyperplasia and mast cell infiltration in DNCB-induced allergic contact dermatitis in mice.

Herein, Chen et al. reported that Kouyanqing Granule (KYQG) reduces sleep-deprivation-induced symptoms in oral ulcers by regulating neuro-immunoendocrine system, oxidative stress levels, and tryptophan metabolism. Kuai et al. revealed the mechanisms of Taodan granules (TDGs) in inhibiting keratinocyte cell growth and inflammatory responses to suppress psoriasis symptoms in IMQ-induced mice. Apart from the improvement of inflammatory reactions in skin lesions, another research reported by Gan et al. has shown that burn ointment (BO), a traditional Chinese medical formulation, can be used to promote cutaneous wound healing due to its antimicrobial and anti-inflammatory qualities. Meanwhile, phenolic phytochemicals such as xanthones and flavonoids which comprise oxygenated heterocycles structures are well-known antioxidants with multi-targeting mechanisms. Many studies reported that prenylated *α*- and *γ*-mangostin, glucosylated mangiferin and xanthone gambogic acid can be used for treating skin diseases (Gunter et al.). Zhang et al. identified the role of isovitexin, a flavonoid found in the leaf of *Celtis sinensis*, in ameliorating contact dermatitis. Other key metabolites from microalgae including carotenoids, astaxanthine and lutein known to possess anti-inflammation and anti-oxidative properties are also used in the remedy of various skin diseases (Choo et al.). Despite the promising therapeutic effects, natural products were limited in terms of their stability, solubility, and bioavailability. The development of novel delivery approaches, such as micro- and nano-emulsions, nanocarriers, microneedles, and cryogels could help to alleviate these limitations (How et al.; Xie et al.). These strategies could help to achieve the therapeutic effect through its delivery to the skin lesions and interact with underlying immune cells and inflammatory mediators.

## Regulating Immuno-Pathogenesis of Skin Diseases–Pathways and Mechanisms

Inflammatory skin diseases are mostly characterized by skin lesions with systemic manifestations. The immune system plays a crucial pathogenetic role. Mechanistically, immune mediators that trigger molecular changes in the local tissue cells are driving inflammatory responses (Richmond and Harris, 2014; Schön, 2019). Translational immunology has contributed significantly to the understanding and controlling of inflammatory skin diseases. Therapeutics targeting IL-23 and IL-17 are approved for clinical use and they showed high efficacy (Gaffen et al., 2014; Ghoreschi et al., 2021). The detailed mechanism of natural products in targeting immuno-pathogenesis of skin diseases have been reported.

Herein, the LXJD formula was shown to inhibit keratinocyte proliferation and suppressed CD3^+^ T cells infiltration in IMQ-induced mice. LXJD formula treatment suppressed IL-1β, IL-6, TNF-α, COX2 by regulating several signaling pathways including MAPK, PI3K/AKT, and NF-қB (Zhao et al.). Meanwhile, CBT suppressed serum immunoglobulin E (IgE) levels and pro-inflammatory cytokines (IL-4 and IL-6) and chemokines (IL-8 and CXCL10) expression in DNCB-treated mice with AD-like lesions (Lee et al.; Lee et al.). Mechanistically, CBT activates Nrf2 and HO-1 signaling in HaCaT cells stimulated with TNF-α/IFN-γ. Derma-H, a mixture that contains *Astragalus membranaceus* Fisch. ex Bunge and *Nepeta tenuifolia* Benth, inhibit Th2-mediated cytokines expression and suppress NGF-TrkA signaling, all which improved the skin lesions in DNCB-treated mice (Jo et al.).

It has been reported that KYQG inhibit systemic inflammation by reducing the expression of TNF-α, IL-1β, IL-6, IL-18, and MCP-1 (Chen et al.). BO on the other hand have shown to suppress TNF-α and inhibit VEGF and TGF-β1 levels in the serum. As a wound healing agent, BO also resulted in the increase of collagen-I expression, possibly through regulating the PI3K, AKT, and mTOR signaling pathways (Gan et al.). Interestingly, TDGs attenuate IMQ-induced lesions in a psoriatic mouse model and were shown to reduce IL-17a, TNF-α, and NF-κB expression, and upregulation of the Gly- Ser-Thr metabolism axis (Kuai et al.). Isovitexin decrease proinflammatory cytokines (TNF-α, IFN-γ, IL-2 and IL-17A) expression, and modulated the MAPK and STAT signaling pathways as well as SHP2 phosphorylation (Zhang et al.).

## Systematic Experimental Pharmacological Evaluation Approaches of Natural Products

To elucidate the mechanistic pathways targeted by phytochemicals and herbal mixtures, a systematic experimental pharmacological evaluation approach is needed. Natural products which comprise herbal formulation mixtures are most often based on their theory of medicine with multiple ingredients interactions. The complex herbal mixtures along with their pharmacological activity could complicate the understanding of their mechanisms of action. Chemical profiling based on UPLC-MS analysis is the most often used approach in identifying chemical ingredients of natural products extracts and formulations, followed by experimental evaluation of their pharmacological activities in cellular and animal models. Systems pharmacology can be used as an approach to integrate data on oral bioavailability, drug targets prediction and validation, as well as disease network pharmacology. Recent studies have reported the use of *in silico* ADME (absorption, distribution, metabolism, and excretion) model to select active agents with favorable pharmacokinetic properties (Ru et al., 2014; Yuan et al.). In addition, in-depth metabolomic profiling serves to provide insights into the biological targets regulated by natural products. Gan et al. combined the use of pharmacological and metabolomics approaches in identifying the biomarkers of KYQG. It was highlighted that identification and quantification of absorbed chemicals and its metabolites should be performed to correlate biomarkers targeted in response to their corresponding active constituents.

## Conclusion

In summary, this Research Topic enhances our knowledge on recent exciting progress in the field of molecular pharmacology of natural products in skin diseases. Herbal formulations and their active constituents can improve inflammatory skin lesions in animal models and human disease patients, and they regulate pro-inflammatory cytokines expression and its corresponding signaling pathways. Furthermore, the combination of technologies between chemical profiling, metabolomics and pharmacodynamics will continue to uncover the mechanisms and regulatory pathways of natural products in targeting human skin diseases.
